# Lack of intergenerational reproductive conflict, rather than lack of inclusive fitness benefits, explains absence of post-reproductive lifespan in long-finned pilot whales

**DOI:** 10.1093/beheco/arad062

**Published:** 2023-08-29

**Authors:** Jack L McCormack, Kevin Arbuckle, Karen Fullard, William Amos, Hazel J Nichols

**Affiliations:** Department of Biosciences, Swansea University, Swansea SA2 8PP, Swansea, UK; Department of Biosciences, Swansea University, Swansea SA2 8PP, Swansea, UK; Department of Radiology, Royal Prince Alfred Hospital, Missenden Road, Sydney, New South Wales 2050, Australia; Department of Zoology, University of Cambridge, Downing Street, Cambridge CB2 3EJ, Cambridgeshire, UK; Department of Biosciences, Swansea University, Swansea SA2 8PP, Swansea, UK

**Keywords:** cetaceans, costs vs. benefits, demography, post-reproductive lifespan, social structure

## Abstract

Life-history theory suggests that individuals should reproduce until death, yet females of a small number of mammals live for a significant period after ceasing reproduction, a phenomenon known as post-reproductive lifespan. It is thought that the evolution of this trait is facilitated by increasing local relatedness throughout a female’s lifetime. This allows older females to gain inclusive fitness through helping their offspring (known as a mother effect) and/or grandoffspring (known as a grandmother effect), rather than gaining direct fitness through reproducing. However, older females may only benefit from stopping reproducing when their direct offspring compete with those of their daughters. Here, we investigate whether a lack of post-reproductive lifespan in long-finned pilot whales (*Globicephala melas*) results from minimal benefits incurred from the presence of older females, or from a lack of costs resulting from mother–daughter co-reproduction. Using microsatellite data, we conducted parentage analysis on individuals from 25 pods and find that younger females were more likely to have offspring if their mother was present in their pod, indicating that mothers may assist inexperienced daughters to reproduce. However, we found no evidence of reproductive conflict between co-reproducing mothers and daughters, indicating that females may be able to reproduce into old age while simultaneously aiding their daughters in reproduction. This highlights the importance of reproductive conflict in the evolution of a post-reproductive lifespan and demonstrates that mother and grandmother effects alone do not result in the evolution of a post-reproductive lifespan.

## Teaser

If having more offspring increases evolutionary fitness, why do females of some species, like humans and certain whales, stop reproducing long before they die? Long-finned pilot whales are biologically very similar to species who do this, yet they continue reproducing until death. We show that this species can help boost their offspring’s reproduction without suffering costly competition between mothers and daughters. For “menopause-like” life histories to evolve, the costs may be more important than the benefits.

## INTRODUCTION

Classical life-history theory suggests that early cessation of reproduction should put an individual at a fitness disadvantage, and therefore reproduction should continue until death (Johnstone and Cant 2019). Despite this, females of a handful of mammal species display a widespread and substantial period of post-reproductive lifespan (PRLS) that represents a distinct life stage (Croft et al. 2015; Ellis et al. 2018). In humans, most women stop reproducing by age 38, yet regularly live into their 70s and 80s (Towner et al. 2016). This decoupling of somatic and reproductive senescence, known as menopause in humans, could be influenced by modern medicine, lifestyle, and sanitation (Austad 1994). This trait, however, is also reflected in modern day hunter–gatherer societies which are largely sheltered from modernization. Within the Hadza population of Tanzania, for example, the 40% of women who live past 50 (the average age of menopause in humans) regularly survive into their 70s, indicating that this trait is a distinct feature of human life history (Blurton Jones 2016).

Aside from humans, only four other mammal species are known to display significant PRLS: killer whales (*Orcinus orca*), short-finned pilot whales (*Globicephala macrorhynchus*), beluga whales (*Delphinapterus leucas*), and narwhals (*Monodon monoceros*) (Nielsen et al. 2021), while some evidence for extended PRLS has also been found in false killer whales (*Pseudorca crassidens*) and Asian elephants (*Elephas maximus*) (Photopoulou et al. 2017; Chapman et al. 2019). Although the kinship dynamics of some of these species is poorly understood, long-term studies of resident killer whales (Croft et al. 2017) and humans (Johnstone and Cant 2008), combined with theoretical work (Cant and Johnstone 2008; Johnstone and Cant 2010), suggest that the evolution of substantial post-reproductive lifespan may be constrained to species where local relatedness increases throughout a female’s lifetime. In humans, increasing relatedness may be the result of female-biased dispersal, whereby historically women tended to disperse to a new group prior to reproduction and hence had low relatedness to their group, but local relatedness later increases as they produce their own children (Johnstone and Cant 2008). Killer whales, and potentially other toothed whales that display extended PRLS, display bisexual philopatry but non-local mating (Rendell et al. 2019); a pattern that also leads to an increase in relatedness over the lifespan as philopatric offspring accumulate in the social group (Johnstone and Cant 2010). In situations where local relatedness increases with age, older females might gain greater inclusive fitness via helping their offspring and/or grandoffspring to survive and reproduce, rather than by reproducing themselves (Reznick et al. 2005).

Strong evidence for post-reproductive females aiding the survival of their adult offspring (known as mother effects) has been found in killer whales, where mortality risk of adult offspring (over 30 years old) increases 13.9-fold in sons and 5.4-fold in daughters the year after the death of a post-reproductive mother (Foster et al. 2012). Similarly, there is evidence that older females may be able to boost the fitness of their grandoffspring. These “grandmother effects” are well studied in human populations. Data collected from 18th and 19th century population census records in Finland has revealed that the presence of a maternal or paternal post-reproductive grandmother significantly increased the lifetime reproductive success of grandoffspring (Lahdenperä et al. 2004). Furthermore, data from pre-industrial French settlers in Quebec have shown that such grandmother effects decrease with increased geographical distance between grandmother and daughter, indicating that the physical presence of a post-reproductive grandmother can increase the fitness of her grandoffspring (Engelhardt et al. 2019). Such grandmother effects have also been observed in killer whale societies, where the survival benefits provided by non-reproductive grandmothers surpass those provided by reproductive grandmothers (Nattrass et al. 2019).

Aid from post-reproductive females is thought to come in a variety of forms, such as the sharing of food and ecological knowledge (Péron et al. 2019). In resident killer whale societies, which are heavily reliant on chinook salmon (*Oncorhynchus tshawytscha*), post-reproductive females are known to lead their pods to foraging grounds which vary considerably in space and time. Such leadership has been shown to be particularly strong during periods of low salmon abundance, suggesting that post-reproductive females act as repositories of ecological information (Brent et al. 2015). It is unclear, however, whether the same pattern exists in other cetaceans (Nattrass et al. 2019).

The mere fact that older females are able to increase the fitness of their descendants does not guarantee the evolution of PRLS. Reproductive female African elephants (*Loxodonta africana*), for example, are known to actively engage in the survival of their grandoffspring by acting as repositories of ecological information (Moss and Lee 2011). Furthermore, mother presence is shown to increase the reproductive success of adult sons in bonobos (*Pan paniscus*) despite females remaining reproductive throughout their lifetime (Surbeck et al. 2011). This suggests females are only likely to cease reproduction if it comes at a cost to kin. The reproductive conflict hypothesis suggests that older females might halt reproductive activity to avoid intergenerational competition with their daughters, which may reduce total fitness when losses of inclusive fitness are not compensated by direct fitness gains (Cant and Johnstone 2008). Evidence for severe intergenerational conflict between non-related individuals has been found in pre-industrial Finnish society, where the simultaneous birth of offspring by consecutive generations of in-laws reduced offspring survival by up to 66% (Lahdenperä et al. 2012). However, this study found no evidence for reproductive conflict between mother–daughter pairings. Conversely, evidence for such conflict has been found in resident killer whale societies, where the mortality risk associated with co-breeding is 1.7 times higher in calves born to older generation females than those born to younger generation females (Croft et al. 2017).

Despite having similar social structures and relatedness patterns to their sister species (the short-finned pilot whale, which shows a substantial PRLS), female long-finned pilot whales (*Globicephala melas*) show a gradual reduction in reproductive rate with age rather than the complete somatic senescence (Nielsen et al. 2021). Both species display bisexual philopatry and non-local mating, with groups being comprised of numerous related matrilines (Foote 2008; Johnstone and Cant 2010; Boran and Heimlich 2019; Nichols et al. 2020). Female long-finned pilot whales have been shown to become increasingly related to their pod members throughout their lifetime, which might be expected to predispose the species to evolving PRLS (Nichols et al. 2020). Furthermore, previous work on this study system showed that females are less likely to be pregnant when they have more daughters (but not sons) present in their pod (Nichols et al. 2020). This suggests that mother–daughter co-reproduction could potentially impose a cost to continued reproduction by the mothers, but it remains unclear whether such a cost is realized. The lack of a widespread PRLS in long-finned pilot whales may therefore result from either a lack of benefits to helping descendent kin (minimal mother and grandmother effects) or from a low cost of mothers co-breeding alongside their daughters (minimal reproductive conflict). If long-finned pilot whales benefit from helping descendant kin and incur significant costs associated with mother–daughter co-breeding, then a third possibility is that the evolution of a PRLS is ongoing and is yet to represent a distinct life stage.

Here, we use physiological and genetic data from 1375 long-finned pilot whales of 25 pods collected during legal subsistence hunts between 1986 and 1988 to investigate what underlies the lack of PRLS in this species. We first determined the maximum number of generations in each pod to quantify the degree of intergenerational overlap, thus facilitating the potential for intergenerational fitness benefits and reproductive conflict. Second, to test whether mother and grandmother effects significantly contribute to offspring fitness, we investigated whether the presence of an individual’s mother or grandmother in the pod had a significant effect on three fitness traits: an individual’s size (for their age), the number of offspring females had, and pregnancy status. Finally, to test whether reproductive conflict may reduce offspring fitness, we tested whether offspring born into potential intergenerational reproductive conflict were significantly smaller than those not born into intergenerational conflict. Given that long-finned pilot whales do not display PRLS, we predict that either older females do not impart fitness benefits to their offspring or grandoffspring and/or that a lack of costs associated with intergenerational co-breeding allows females to continue reproducing into old age without reducing the fitness of descendant kin.

## METHODS

### Data collection

Data for this study originate from drive fisheries in the Faroe Islands, where researchers commissioned by the International Whaling Commission and the United Nations Environmental Programme conducted a survey of the local long-finned pilot whale population in a study initiated by the Faroese Government (Bloch et al. 1993). The hunts, which were opportunistic in nature, involved the sightings of entire pods from land or boat which were driven into whaling bays for slaughter (Zachariassen 1993). Pods are known to be highly cohesive, meaning that almost all pods were sampled in their entirety (Amos et al. 1993).

The initial dataset was comprised of 1804 individuals, of which 1057 (58.59%) were female, 703 (38.97%) were male and 44 (2.44%) were unsexed. This biased sex ratio is likely a result of females having longer lifespans than males (Bloch et al. 1993). Our samples were collected from 25 pods, of which 3 pods (pod IDs 323, 819 and 829) were partially sampled due to the separation of pods during hunting (Bloch et al. 1993). The size of pods ranged from 17 to 194 individuals (median = 63). Each analysis used a different subset of these individuals (e.g., when data were restricted by age and/or sex), which is further explained in the statistical methods.

### Body length, sex, and age measurements

Upon capture, each individual was given a unique identity number and their length and sex were recorded. Body length was measured following the standard of Norris (1961), taking measurement to the nearest cm from the foremost part of the skull to the fluke notch, running parallel to the spine. To assess the reliability of length measurements, repeats were taken for 50 whales three times. Repeated measurements all varied within ≤ 5% of total length (Bloch et al. 1993). Sex was determined via macroscopic observation. The reproductive status of females was recorded during on-site dissection, and therefore, all pregnancies were noted (Bloch et al. 1993).

Where possible, age was calculated using growth rings in cementum and dentine in teeth. The tip of the lower mandible was severed and then allowed to rot in a heat pressured cabinet for 2 weeks before teeth were extracted. Two teeth were mounted onto a wooden block with epoxy resin and bisected longitudinally using a diamond rotating saw, allowing for growth layers to be counted (Lockyer and Desportes 1987; Bloch et al. 1993). Due to time constraints, age data for 250 individuals (13.86%) was not obtained using this method. For individuals that had length data available (*N* = 170, 9.86% of individuals with age data), Nichols et al. (2014) used quadratic regression of age against length to estimate age. Due to the rapid growth exhibited in the early lives of long-finned pilot whales, which is proceeded by a plateau in later life, this method was only applicable to individuals prior to reaching maturation. Therefore, females larger than 400 cm were assumed to be 10 years or older, and males larger than 500 cm were assumed to be 15 years or older. Ten individuals had no age or length data noted. Individuals that had their age estimated based on their length were not included in analyses for which length was the response variable. Ages range from 0 (representing the 154 unborn fetuses which were dissected from mothers upon capture) to 55 years.

### Genetic data

Skin samples were taken from the posterior of the dorsal fin and tissues were preserved either at −20 °C or using a 20% DMSO solution saturated with NaCl (Bloch et al. 1993). A modified Chelex extraction protocol was used for DNA extraction (Walsh et al. 1991). Tissues were incubated overnight in a 320 µL extraction buffer, and were then centrifuged at 10,000*g* for 2 min, before 200 µL of supernatant was transferred to 200 µL of buffered Chelex solution (Fullard et al. 2000). Genotyping was attempted for a core set 9 microsatellite primer pairs: 199/200, 409/470, 415/416, 417/418, 464/465, 468/469 (Amos et al. 1993), EV37, EV94, EV1 (Valsecchi and Amos 1996). In subsequent studies, 809 individuals in the original dataset were genotyped at a further 7 markers: D14, D22 (Shinohara et al. 1997), FCB 6/17, FCB3, FCB1 (Buchanan et al. 1996), SW10 (Richard et al. 1996), and GM8 (Fullard et al. 2000).

### Maternity assignment

The maternal assignment analysis was carried out following a similar analysis by Nichols et al. (2020) using Cervus (Marshall et al. 2003), a computer program which uses microsatellite data and likelihood to assign parentage. We did not carry out a paternity analysis as long-finned pilot whales show extra-pod mating, with offspring remaining with their mother’s pods, meaning fathers were not present (Amos et al. 1993; Allen 2020). Females were considered to be potential mothers if they were at least 6 years older than the offspring in question, as this is when females start to reach sexual maturity (Anabella et al. 2017). Furthermore, females were only listed as potential mothers if they were in the same pod as offspring, as long-finned pilot whales display bisexual philopatry (Möller 2011), and a previous study using the same dataset found that there was no evidence of dispersal between pods (Allen 2020). Twenty-nine individuals were old enough not to have any candidate mothers according to our criteria. Fetuses could be assigned to mothers they were dissected; however, these relationships were confirmed genetically in order to exclude the possibility of field errors. Individuals with less than seven loci typed (118 individuals) were also removed from the analysis to ensure a more reliable maternity assignment (Marshall et al. 2003). Not all individuals would have had their mother sampled, mostly through the mother having already died, but also through the incomplete sampling of a pod. In addition, we cannot exclude the possibility that individuals either evaded capture or emigrated from the pod prior to being hunted (Nichols et al. 2020). Therefore, for the Cervus analysis, it was estimated that 50% of mothers had been sampled. An allele frequency analysis calculated that the proportion of loci genotyped was 0.73, while the average per-allele error rate was 0.01. These values were incorporated into simulations to calculate critical delta, the threshold probability used to assign parentage. No assumptions were made regarding the relatedness of candidate mothers to true mothers, as a previous study on this dataset had shown average relatedness between females in the same pod to be low (Nichols et al. 2020), such that candidate and true mothers share on average a sufficiently low proportion of genetic material to maintain the reliability of assignments. An Identity analysis indicated that one individual had been sampled twice, so one replicate of this individual was removed from the dataset.

### Statistical methods

Statistical analyses were carried out using RStudio version 1.1.463 (RStudio Team 2015). The package “lme4” (Bates et al. 2015) was used to conduct a series of general linear mixed models (GLMMs). The identity of the pod that each individual was sampled from was added as a random factor for each analysis to account for any unknown variance between pods. Model assumptions were checked using the R package “DHARMa” (Harting 2021). *P* values were calculated using likelihood ratio tests, and nonsignificant interactions were removed from the models. All main effects were retained regardless of significance.

### Investigating potential mother and grandmother effects

**Figure 1:**
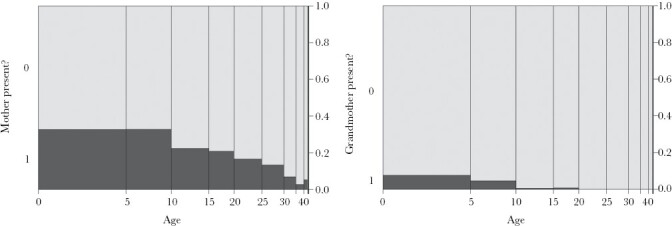
Changes in the proportion of individuals with mothers (a) and grandmothers (b) present with age. Analyses for grandmother effects were studied in individuals under 20 as no individuals above this age had a mother present, and mother effects were studied in individuals under 35, as only a small proportion of individuals above this age had mothers present. Bar width represents the number of individuals in each 5-year age bracket.

We investigated whether there was evidence of fitness benefits to adult offspring resulting from the presence of mothers or grandmothers in the pod. We investigated three fitness traits: (1) length, (2) the number of offspring genetically assigned to females, and (3) the pregnancy status of females at time of capture. These metrics for fitness are similar to those used by previous studies using the same dataset (Nichols et al. 2014, 2020). Unlike studies investigating mother and grandmother effects in killer whales (Croft et al. 2017; Nattrass et al. 2019), longitudinal data collected across multiple decades does not exist for long-finned pilot whales, so direct estimates of mortality risk over the lifespan cannot be made from this species. Older individuals were less likely to have their mother or grandmother present in the pod (Figure 1), due to mortality of older females. We therefore restricted analyses analysis of mother effects to individuals under 35 years old and analysis of grandmother effects to individuals under 20 years old (note that the presence of a mother was also included in models of grandmother effects). The typical gestation period for long-finned pilot whales is 15–16 months, with a female having an average of 9 offspring throughout her lifetime. Lactation typically continues for 22 months after birth, resulting in a 40-month reproductive cycle. The average breeding interval in long-finned pilot whales is therefore 3.3 years (Martin and Rothery 1993).

#### Are whales larger for their age if their mother or grandmother is present?

To investigate the effects of the presence of a mother or grandmother on the size of offspring, we fitted GLMMs with Gaussian error distribution with length as the response variable and mother presence, grandmother presence, age, age^2^, and sex as fixed effects. Sex was added as an interaction with age, age^2^, and mother and grandmother presence to account for sex-specific growth rates and the likely disproportionate investment in male offspring required to facilitate such differences (Nichols et al. 2014). Note that in all models age was fitted as both a linear and quadratic term to account for a more rapid growth rate exhibited by long-finned pilot whales in early life which slows after an individual stops suckling (Alvarado-Rybak et al. 2019). These analyses did not include fetuses, individuals that were unsexed or individuals that had their age calculated from their length. This left a final dataset of 1222 individuals for the analysis investigating effects in whales under the age of 35 (of which 726 [59.4%] were female and 496 [40.6%] were male) and 909 individuals in the analysis investigating effects in whales under the age of 20 (of which 510 [56.1%] were female and 399 [43.9%] were male).

#### Do females have more offspring genetically assigned to them if their mother or grandmother is present in their pod?

To test whether females had more existing offspring for their age if their mother or grandmother was present, we fitted GLMMs with Poisson error distribution, with the number of offspring as the response variable and age, age^2^, mother presence, and grandmother presence as fixed effects.

Most individuals under the age of 20 only had one offspring present. Therefore, we ran another model investigating the effect of mother and grandmother presence on the probability that individuals had offspring present. We constructed a binomial GLMM with offspring presence (coded as 1/0) as the response variable, and age, age^2^, mother presence, and grandmother presence as fixed effects. For all analyses of reproductive fitness, females below reproductive age (6 years) were excluded, as were females without a precise age (those without age data from tooth sections or that were estimated to be over 10 years old based on their length). This left a final dataset of 294 individuals in analyses studying effects in females under 20 and 510 individuals in analyses studying the effects in females under 35.

#### Are females more likely to be pregnant if their mother or grandmother is present in their pod?

To investigate whether females were more likely to be pregnant if their mother or grandmother was present, a GLMM with binomial distribution was fitted with pregnancy as the response variable and age, age^2^, mother presence, and grandmother presence as fixed effects. For both analyses looking at the effects of mother and grandmother presence on reproductive conflict, age was scaled (by subtracting the mean and dividing by the standard deviation) to avoid singularity issues during model fitting. This analysis used the same dataset as our models of number of offspring (immediately above), consisting of 294 females under 20 and 510 females under 35.

### Is there any evidence for intergenerational reproductive conflict?

To determine whether offspring growth was affected by reproductive conflict, a GLMM with Gaussian error distribution was fitted with offspring length as the response variable and age, age^2^, sex, and whether the mother was in reproductive conflict (coded as 1/0) as the fixed terms. In this instance, reproductive conflict was defined as occurring when a mother gave birth to offspring within 2 years of her daughter (or vice versa). Two years was selected to cover the period when females need to acquire energy for mate competition, gestation and birth, this being the period of most intense conflict. The same approach was used by Croft et al. (2017) when investigating the effects of reproductive conflict in killer whales. Sex was added as an interaction with age, age^2^, and reproductive conflict to account for differences in sex specific growth rates and the possible impact of disproportionate investment in sons on reproductive conflict. This analysis was restricted to individuals under 9 years old as the oldest individual known to be born into reproductive conflict was 8 years old. Fetuses were excluded from the analysis, as were unsexed individuals (since growth rates are sex-specific) and individuals with age calculated through quadratic regression were removed because length was the response variable. The final dataset for these analyses included 565 individuals, of which 290 (51.3%) were female and 275 (48.7%) were male.

### Ethical note

Data were collected from dead whales during non-commercial subsistence hunts carried out between 1986 and 1988 in the Faroe Islands. The Faroese government approved this data collection, and no financial transaction was made for access to whale carcasses or genetic samples, therefore, this study did not contribute to the trade or consumption of whale meat. Full details of these hunts can be found in Bloch et al. (1993). The IUCN classify the conservation status of the long-finned pilot whale as “Least Concern” (Minton et al. 2018). This study was approved by Swansea University College of Science Ethics Committee (approval number: SU-Ethics-Student-200721/3672.)

## RESULTS

**Figure 2:**
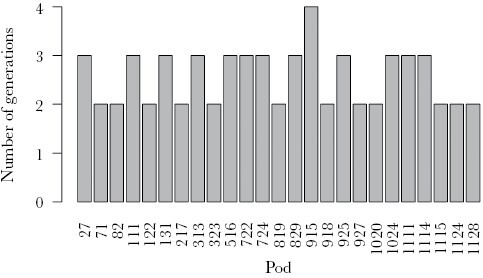
The maximum number of generations within pods.

**Figure 3:**
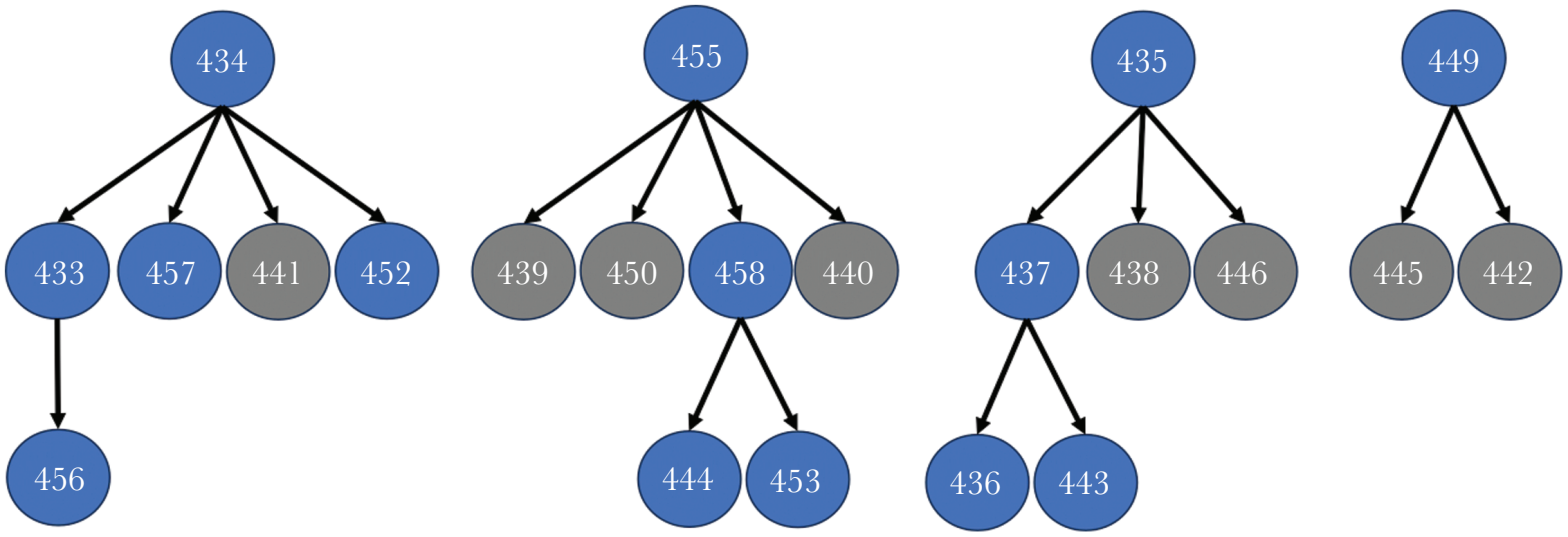
An example of the typical kin relationships found in a pod (from pod 131). Numbers on nodes represent an individual’s ID number. The pod is composed of multiple matrilines, with four older females with their offspring and grandoffspring. Blue nodes represent females whilst grey nodes represent males. This pedigree represents 22 out of the 26 individuals in this pod; 4 individuals were without assigned offspring or an assigned mother (three females aged 8, 10, and 12, and one male aged 15).

We included 1459 offspring in the Cervus analysis, of which 375 (27.3%) were assigned mothers at a confidence level of ≥ 95% and a further 459 (33.4%) at a confidence level of ≥ 90%, leaving 916 (66.6%) individuals without an assigned mother. To remain conservative, subsequent statistical analyses were restricted to assignments made at ≥ 95% confidence. Younger individuals were more likely to be assigned mothers and grandmothers (Figure 1), which is consistent with increasing probability of mother and grandmother death with age. A large proportion of younger individuals, however, are unassigned, which could result from insufficient genetic resolution and/or from some mothers remaining unsampled, perhaps due to the temporary fission of pods, as has been observed in some populations (Augusto et al. 2017b). The maximum number of generations within pods ranged from 2 to 4 (Figure 2) and grandmothers were present alongside their daughters and grandoffspring in 13 of 25 pods (52%). Pods were composed of several related matrilines (an example is provided in Figure 3), giving both mothers and grandmothers the opportunity to aid descendant kin.

### Are whales larger for their age if their mother or grandmother is present?

There was no evidence that individuals were larger for their age if their mother (individuals under 20 GLMM: χ^2^ = 0.56, df = 1, *P* = 0.454, Table 1; individuals under 35 GLMM: χ^2^ = 2.58, df = 1, *P* = 0.108, Table 1) or grandmother (individuals under 20 GLMM: χ^2^ = 0.05, df = 1, *P* = 0.826, Table 1) was present in the pod. As expected, sex-specific growth rates were found in interactions between age and sex as a predictor of length.

**Table 1:** A summary GLMMs carried out

Model	Term	Effect size	Std. Error	*t*/*z* value	*P*-value
Are individuals under 20 larger for their age if mother/grandmother is present?	Intercept	219.759	3.200	68.68	NA
(Response variable: length, number of individuals: 909, number of pods: 25.)	Age	27.358	0.717	38.18	NA
	Age²	−0.881	0.038	−23.03	NA
	Sex	4.442	3.965	1.12	NA
	Mother	−1.520	2.012	−0.76	0.454
	Grandmother	−0.901	4.048	−0.22	0.826
	Age × Sex	2.559	1.112	2.30	0.021
	Age² × Sex	0.165	0.061	2.73	0.006
Are individuals under 35 larger for their age if mother present?	Intercept	242.868	2.925	83.04	NA
(Response variable: length, number of individuals: 1222, number of pods: 25.)	Age	18.452	0.357	51.64	NA
	Age²	−0.398	0.011	−34.81	NA
	Sex	−7.051	3.290	−2.14	NA
	Mother	−2.922	1.811	−1.61	0.108
	Age × Sex	7.318	0.573	12.77	<0.001
	Age² × Sex	−0.089	0.018	−4.89	<0.001
Do females under 20 have more offspring for their age if mother/grandmother present?	Intercept	−1.936	0.260	−7.45	NA
(Response variable: number of offspring, number of individuals: 294, number of pods: 25.)	Age	1.260	0.255	4.93	<0.001
	Age²	−0.525	0.181	−2.90	0.001
	Mother	0.603	0.275	2.19	0.030
	Grandmother	−0.540	1.049	−0.52	0.579
Do females under 35 have more offspring for their age if mother is present?	Intercept	−1.145	0.177	−6.46	NA
(Response variable: number of offspring, number of individuals: 510, number of pods: 25.)	Age	0.960	0.111	8.66	<0.001
	Age²	−0.266	0.077	−3.43	<0.001
	Mother	0.040	0.170	0.23	0.819
Are females under 20 more likely to have offspring present if grandmother/mother present?	Intercept	−2.093	0.363	−5.76	NA
(Response variable: whether individual has offspring present, number of individuals: 294, number of pods: 25.)	Age	1.633	0.320	5.11	<0.001
	Age²	−0.549	0.244	−2.25	0.016
	Mother	0.998	0.416	2.40	0.018
	Grandmother	−0.756	1.470	−0.51	0.602
Are females under 20 more likely to be pregnant if mother/grandmother present?	Intercept	−0.801	0.220	−3.64	NA
(Response variable: pregnancy status, number of individuals: 294, number of pods: 25.)	Age	0.697	0.206	3.39	<0.001
	Age²	−0.738	0.200	−3.69	<0.001
	Mother	−0.221	0.385	−0.57	0.562
	Grandmother	1.676	1.054	1.59	0.135
Are females under 35 more likely to be pregnant if mother is present?	Intercept	−1.060	0.210	−5.06	NA
(Response variable: pregnancy status, number of individuals: 510, number of pods: 25.)	Age	0.097	0.134	0.72	0.471
	Age²	−0.493	0.142	−3.47	<0.001
	Mother	−0.021	0.284	−0.07	0.942
Does reproductive conflict affect the size of individuals under 5?	Intercept	200.723	3.883	51.69	NA
(Response variable: length, number of individuals: 353, number of pods: 25.)	Age	47.468	3.370	14.09	<0.001
	Age²	−4.429	0.815	−5.44	NA
	Sex	2.030	3.875	0.52	NA
	In conflict	3.785	6.406	0.59	0.545
	Age² × Sex	1.162	0.419	2.77	0.00540
Does reproductive conflict affect the size of individuals under 9?	Intercept	200.677	3.341	60.07	NA
(Response variable: length, number of individuals: 565, number of pods: 25.)	Age	38.120	1.524	25.01	<0.001
	Age²	−1.879	0.188	−10.02	<0.001
	Sex	15.935	2.086	7.64	<0.001
	In conflict	2.010	4.963	0.41	0.678

Effect sizes, standard error, and *t*/*z* values were taken from GLMM results, and *P* values were calculated using Likelihood Ratio Tests. *P* values are not included for main terms which were contained within significant interactions.

### Do females have more offspring genetically assigned to them if their mother or grandmother is present in their pod?

**Figure 4:**
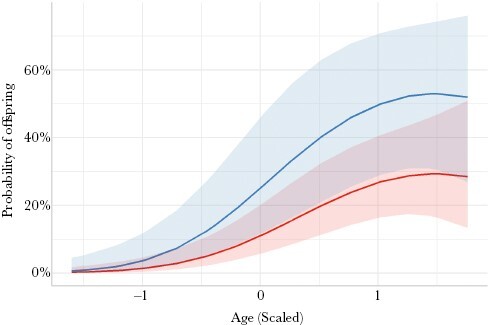
Females with a mother present within the pod (blue) were more likely to have offspring present than those without (red). Lines represent the model predictions of the probability that a female will have at least one offspring assigned throughout her lifetime and bands represent standard error.

Females under 20 had more offspring present (GLMM: χ^2^ = 4.69, df = 1, *P* = 0.030 Table 1) and were more likely to have at least one offspring assigned (GLMM: χ^2^ = 5.65, df = 1, *P* = 0.018, Figure 4, Table 1) if their mother was present in their pod. However, the presence of a grandmother in the pod had no impact on offspring number (GLMM: χ^2^ = 0.31, df = 1, *P* = 0.579, Table 1) or presence (GLMM: χ^2^ = 0.27, df = 1, *P* = 0.602, Table 1). When the analysis was expanded to all females under 35, there was no significant impact of mother presence on the number of offspring assigned to a female (GLMM: χ^2^ = 0.05, df = 1, *P* = 0.819, Table 1).

### Are females more likely to be pregnant if their mother or grandmother is present in their pod?

Females were not more likely to be pregnant if their mother (GLMM: χ^2^ = 0.01, df = 1, *P* = 0.942, Table 1) or grandmother (GLMM: χ^2^ = 2.24, df = 1, *P* = 0.135, Table 1) were present in their pod.

### Is there any evidence for intergenerational reproductive conflict?

We found no evidence that offspring were smaller for their age if they were born into potential reproductive conflict (GLMM: χ^2^ = 0.17, df = 1, *P* = 0.678, Table 1). It is possible that the effects of reproductive conflict may be greater in younger individuals (where the period of conflict had been more recent). To investigate this possibility, we ran this model again for individuals under 5, and results were concordant with the original model (GLMM: χ^2^ = 0.37, df = 1, *P* = 0.545, Table 1).

## DISCUSSION

We investigated why long-finned pilot whales may lack substantial PRLS, despite having a similar relatedness structure to closely related species that do have a prolonged post-reproductive period. Specifically, we tested the predictions that a lack of PRLS is due to a lack of fitness benefits resulting from mother and grandmother effects, and/or is a result of low levels of intergenerational reproductive conflict. We found evidence that mothers benefit their daughters; young adult females (aged between 6 and 20 years old) were more likely to have offspring present in their pod and had more offspring present if they had a genetically assigned mother in their pod. However, unlike mothers, grandmothers had no detectable fitness benefit to their grandoffspring. We also found no evidence of intergenerational reproductive conflict; offspring born into conflict were no smaller than offspring born outside of conflict. Together, these results suggest that long-finned pilot whales benefit from the presence of older females in the pod without suffering the costs of intergenerational conflict when older females co-reproduce with their daughters. This may explain the lack of PRLS in long-finned pilot whales, despite this trait occurring in some of their close relatives.

While we found that mothers appear to benefit their adult daughters aged under 20, we did not find the same benefits when this analysis was extended to include daughters aged up to 35 years old. This suggests that mothers may help younger, inexperienced daughters to raise offspring and/or facilitate their daughters’ first reproduction at an earlier age. It is possible that older females engage in alloparental care such as allonursing, whereby females suckle young of other females. Allonursing has been observed in other odontocetes, such as sperm whales (*Physeter macrocephalus*) and captive beluga whales (*Delphinapterus leucas*) (Leung et al. 2010; Konrad et al. 2019). Although this behavior has not been observed in long-finned pilot whales, the collection of such data is challenging as it is extremely difficult to observe suckling or to identify maternal and grandmaternal relationships in the field (Augusto et al. 2017a). Alternatively, mothers could babysit the offspring of their daughters, allowing for younger females to hunt and acquire more energy to offset the costs associated with raising offspring at a younger age. Individuals unlikely to be the mother have been observed to “escort” young in long-finned pilot whales (Augusto et al. 2017a) although such care doesn’t seem to be limited to older females so its relevance to PRLS is unclear. It is also possible that these alloparental benefits are not specifically directed toward younger offspring, but the capacity of mothers to help their daughters is reduced with age as the cost of their own reproduction increases. Finally, it is possible that these potential mother effects could be generated if age at first birth is genetically heritable. In this case, females who have their first offspring at an early age may be more likely to be alive and present in the pod when their daughters start to reproduce. Significant heritability of age at first reproduction has been found in some other cases, including captive butterflies (Saastamoinen 2008) and baboons (Williams-Blangero and Blangero 1995) but not preindustrial humans (Pettay et al. 2005). Unfortunately, the lack of longitudinal data in our study means that we are unable to determine the degree to which reproductive traits are heritable. However, future long-term studies of long-finned pilot whales, including behavioral and genetic data may be able to determine the mechanisms through which young adult daughters have greater reproductive success when their mothers are present in their pod.

We did not find the presence of a grandmother to have a significant effect on the size or reproductive fitness of grandoffspring. In some species, such as killer whales and African elephants, grandmothers play an important role as “repositories” of ecological information which is learnt throughout their lifetime (McComb et al. 2011; Brent et al. 2015). This allows older females to lead groups to resources which vary spatially and temporally, and therefore increase the survival rates of grandoffspring (Greve et al. 2009). If this occurs in long-finned pilot whales, it is likely that all younger individuals within the pod will equally benefit from older female’s ecological knowledge as this behavior cannot be directed at specific individuals, explaining why there are no size or reproductive fitness differences in individuals with and without a grandmother present in their pod. The extent to which the presence of older females might benefit younger individuals is also dependant on the timeframe over which ecological knowledge is acquired. If the majority of knowledge is acquired during the early years of life, then the benefits associated with the presence of older females are likely to be minimal. However, if knowledge is gained throughout an individual’s life in a linear fashion, then the value of having older females present is likely to be greater. Conversely, it is possible that grandmothers are able to direct help toward their grandoffspring, but their help contributes to the survival of these individuals (which was not measured in our study), rather than to their size or reproductive output. This possibility fits with our finding that the presence of a mother in the pod is associated with higher reproductive output of their daughters.

Finally, we found no evidence of intergenerational reproductive conflict; offspring born into potential intergenerational conflict were not smaller than those that weren’t, suggesting that levels of reproductive conflict may be low in this species. This is in alignment with Johnstone and Cant’s (2010) model of the evolution of post-reproductive lifespan, which suggests that early reproductive cessation will only be selected for if inclusive fitness gained by reducing competition with kin outweighs the fitness cost of foregoing reproduction. A lack of intergenerational conflict could therefore explain why the long-finned pilot whale has not evolved a substantial post-reproductive period. A similar situation is seen in African elephants (Moss and Lee 2011), where inter-generational co-reproduction may even be beneficial (Lee et al. 2016). Mother-daughter co-breeding has also been shown to positively influence offspring survival in lions (*Panthera leo*) through alloparenting (Pusey and Packer 1994; Packer et al. 1998) and in bushy tailed woodrats (*Neotoma cinerea*), possibly resulting from the sharing of den sites and other resources (Moses and Millar 1994).

It is possible that the apparent lack of grandmother effects or reproductive conflict is due to limitations in our dataset. Unfortunately, we have no longitudinal data, meaning our dataset reflects the group structure at one specific point in time rather than the long-term relationships within the pod. Therefore, we were not able to investigate the potential impacts of older females on survival or reproductive fitness traits. Similarly, the timing of maternal or grandmaternal death or disappearance is unknown and we are not able to assess the importance of pod stability or the frequency of fission–fusion events (which may occur in this species (Augusto et al. 2017b)), both of which may influence the amount of help mothers are able to provide to their adult daughters. Furthermore, length (which was used as a proxy for fitness) may not be the best indicator for an individual’s fitness, as this is unlikely to change once an individual reaches full maturity. If the data were available, weight may be a better indicator of fitness as this can fluctuate throughout an individual’s lifetime depending on resource availability. We were also unable to assign maternity to all individuals, so some individuals that have mothers or grandmothers present are likely to be incorrectly recorded as not having these relatives present. This will likely reduce our ability to detect impacts of having older female relatives present in the group, although our large sample size should to some degree compensate for this.

A previous study using the same dataset found that fecundity was reduced in older females when they had adult daughters present in the pod (Nichols et al. 2020), suggesting that they may be less likely to reproduce when their offspring are likely to come into conflict with those of their daughters. This suggests that reproductive conflict may occur, but manifests in traits other than offspring size. However, it is also possible that reproductive conflict is genuinely low in this species; long-finned pilot whales have a sub-polar distribution, as opposed to their sister species, the short-finned pilot whale, which occurs in tropical and sub-tropical waters (Olson 2009). Sub-polar waters are more productive than those of the tropics and sub-tropics, meaning competition for resources, and therefore intergenerational reproductive conflict, may be less intense (Péron et al. 2019). Furthermore, individuals found in the Faroe Islands are larger than those found in Iceland and Newfoundland (Betty 2019). This implies that resource abundance is particularly high for the individuals in this dataset compared to those distributed elsewhere, suggesting that the greater ecological productivity of the North-East Atlantic may reduce reproductive conflict to particularly low levels within this population. Finally, as long-finned pilot whales live in large pods (median 57 individuals) with low average levels of relatedness (mean *r* = 0.06–0.15 depending on female age; Nichols et al. 2020), it is likely that any costs of co-reproduction are borne largely by non-relatives. This is in contrast with resident killer whales which live in smaller groups (average pod size = 11; Bigg et al. 1990) with higher relatedness (average pedigree *r* = 0.22–0.33; Croft et al. 2017). Through living in larger pods containing distant kin and non-relatives as well as closer relatives, long-finned pilot whale females may be able to minimize the reproductive cost of intergenerational co-reproduction to the fitness of their daughters.

## CONCLUSION

Despite having patterns of dispersal and relatedness that suggest that they might benefit from PRLS, female long-finned pilot whales do not cease reproducing before they die. Instead, it seems that mothers may be able to improve the reproductive success of their young adult daughters, whilst continuing to reproduce alongside their daughters (although the presence of adult daughters somewhat depresses the fertility of older females; Nichols et al. 2020). Such a system may be possible in long-finned pilot whales because they live in large pods that contain clusters of close relatives, but average relatedness across the group is relatively low (Nichols et al. 2020). This may mean that the costs of co-reproduction are spread across the entire group and so are largely borne by non-relatives, whilst the benefits of providing help can be directed toward close relatives (i.e., daughters). This may tip the fitness cost-benefit ratio toward late life co-reproduction, rather than the evolution of a distinct post-reproductive life stage, as is seen in some other toothed whales.

## Data Availability

Analyses reported in this article can be reproduced using the data provided by McCormack et al. (2023).

## Notes

We thank D. Bloch, C. Lockyer, G. Desportes, R. Mouritsen, and K. Hoydal for collection of the original samples from which the data in this paper were collected.
